# Room‐Temperature Gas‐Phase CO_2_‐to‐C_3_ Coupling by a 4*f*‐Aromatic Cluster

**DOI:** 10.1002/anie.6756333

**Published:** 2026-04-14

**Authors:** Feng‐Xiang Zhang, Xiao‐Wang Li, Ning‐Zheng Li, Sheng‐Gui He, Yu‐Chen Zhang, Li‐Li Xing, Donald G. Truhlar, Jia‐Bi Ma

**Affiliations:** ^1^ Key Laboratory of Cluster Science of Ministry of Education Beijing Key Laboratory of Photoelectronic/Electrophotonic Conversion Materials School of Chemistry and Chemical Engineering Beijing Institute of Technology Beijing China; ^2^ Key Laboratory for Structural Chemistry of Unstable and Stable Species Institute of Chemistry, Beijing National Laboratory for Molecular Sciences and CAS Research/Education Center of Excellence in Molecular Sciences Chinese Academy of Sciences Beijing China; ^3^ Center of Basic Molecular Science Department of Chemistry Tsinghua University Beijing China; ^4^ Energy and Power Engineering Institute Henan University of Science and Technology Henan China; ^5^ Department of Chemistry Chemical Theory Center and Minnesota Supercomputing Institute University of Minnesota Minneapolis Minnesota USA

**Keywords:** aromaticity, CO_2_ conversion, praseodymium, Pr─C bond, rare‐earth

## Abstract

Room‐temperature conversion of CO_2_ into value‐added multi‐carbon products (C_3_ or C_3+_ species) remains a major challenge due to poor selectivity and limited C−C coupling pathways. Using mass spectrometry, photoelectron imaging spectroscopy, and density functional calculations, we identify the 4*f*‐metalla‐aromatic anion PrB_2_C_2_^−^, which—despite lacking a C─C bond—exhibits *σ* and *π* double aromaticity involving a 4*f* atom. We show that PrB_2_C_2_^−^ reacts with CO_2_ at room temperature to generate C_3_B_2_O_2_^−^ with a C─C─C backbone. The C_3_‐chain formation occurs in three stages involving two distinct C−C coupling steps enabled by flexible Pr−*X* bonding (*X* = B, C, O) and Pr‐centered electron shuttling. The unique structure of PrB_2_C_2_^−^ directs CO_2_ activation toward C─C─C coupling rather than CO release. These findings deepen the understanding of *f*‐block‐mediated small‐molecule activation and provide a new and tailored route for producing products with C─C bonds via CO_2_ conversion.

## Graphical abstract

We engineered a 4*f* metalla aromatic cluster, PrB_2_C_2_^−^, that enables efficient sequential C−C coupling with CO_2_ at room temperature, yielding a linear C_3_ backbone. This system provides a complete theoretical catalytic cycle and establishes a new design principle for lanthanide mediated CO_2_ activation.

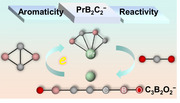

## 1 Introduction

The conversion of CO_2_ into value‐added multi‐carbon products is a promising technology for both CO_2_ recycling and reducing fossil fuel dependence [1, 2, 3, 4]. While C_1_ and C_2_ products (e.g., CO, CH_4_, CH_3_OH, HC(O)OH, CH_2_═CH_2_, and C_2_H_5_OH) [5, 6] have been widely studied, the synthesis of C_3_ or C_3+_ products with higher energy density is even more desirable due to their greater practical utility [2, 7, 8]. In condensed‐phase systems, two CO_2_ hydrogenation pathways have been developed for long‐chain hydrocarbon production [9, 10]. One pathway involves methanol synthesis followed by C−C coupling via the hydrocarbon pool mechanism [11, 12, 13]. The other pathway combines the reverse water gas shift (CO_2_ + H_2_ → CO + H_2_O) with CO‐Fischer–Trosch synthesis [14, 15, 16]. The direct coupling of CO and C_2_ intermediates (such as ethyl and methylcarbonyl) is also widely reported [17, 18]. Recently, Huang et al. employed machine learning to predict key C−C−C coupling pathways for forming C_3_ products [19], and they found that direct C_1_ coupling (*CO + *CH_3_ + *CH_3_ → CH_3_COCH_3_) is more energy‐efficient than C_1_ + C_2_ coupling. However, conventional catalytic processes face significant challenges in producing C_3+_ products; this is primarily due to: (i) competing reaction pathways that diminish C_3_ selectivity [7]; (ii) hindrance of C−C coupling by thermodynamically limited stabilization of *CO intermediates [20]; and (iii) the multi‐metal site requirement that increases preparation complexity [21]. It is crucial to overcome these limitations, for example, by developing innovative strategies.

Lanthanide metals have attracted particular interest in the field of CO_2_ reduction catalysis, because of their unique electronic and structural properties [22, 23, 24]. Lanthanide metals have partially or fully filled 4*f* subshells and multiple favorable oxidation states (ranging from +2 to +4), sometimes leading to intermediate‐energy orbital energies of hybrid orbitals that can accept or donate electrons [23]. Furthermore, their high coordination numbers often endow lanthanide‐based catalysts with remarkable stability [23].

Most studies on lanthanide‐containing aromatic systems primarily focus on La ions, for example, LaB_14,17_^−^ [25], LaC_20_ [26], LaB_8_C_4_^−/0^ [27], La_3_H_9_ [28], LaB_2,3_^−/0^ [29], and La_3_^−^ clusters [30]. Other lanthanide metals are less explored; investigated examples include PrB_2_^−^ [31], ReC_20_ (Re = Ce, Pr, Nd, Pm, Sm, Eu, Gd) [26], and HoB_20_ [32]. Lanthanide boron compounds are especially interesting because the higher orbital energy of boron as compared to carbon allows better energetic matching of boron orbitals with the lanthanide 4*d* orbitals. Given the known polarity and reactivity of metal‐carbon bonds in organolanthanoid complexes [33, 34], lanthanide boron carbide clusters are expected to have unusual electronic structures that make them candidates for unique catalytic roles. A question that arises in this context is aromaticity because, although aromaticity is a key promoter of structural stability, it remains rare in lanthanide metallacycles; but Pr‐containing clusters with B and C provide strong candidates for aromaticity.

In catalytic processes, chemical bond formation typically occurs at a limited number of atomic sites. Consequently, gas‐phase clusters serve as especially promising models for understanding the reactivity of active sites at a strictly molecular level [35, 36, 37, 38, 39, 40, 41, 42, 43]. Numerous gas‐phase clusters have been used to transform CO_2_, including metal ions (e.g., Sc^+^, V_2_^+^, Co_5‐25_^−^) [44, 45, 46], metal hydroxide clusters (e. g., Fe_2_H_1‐3_^−^, PdCuH_4_^−^, NiH^−^) [47, 48, 49], and a diverse set of boron/carbon/nitrogen/oxygen/sulfur‐containing clusters (e.g., YB_1–4_^−·^, Nb_3_C_4_^−^, NbN_2_^−^, Rh_2_VO_1‐2_^−^, TiS^+^) [50, 51, 52, 53, 54]. Building upon this work, our group has also reported the activation of CO_2_ with novel molecular ions, such as ScNH^+^, Nb_2_BN_2_^−^, AuNbBO^−^, and LaB_3,4_O_2_^−^ [55, 56, 57, 58], in addition to the previously mentioned YB_1–4_^−·^, Nb_3_C_4_^−^, and NbN_2_^−^.

Only a few gas‐phase ions are able to produce multi‐carbon products. For instance: (i) the Ta^+^/CH_4_/CO_2_ [59], CuB^+^/CH_4_/CO_2_ [60], and CuTa^+^/CH_4_/CO_2_ [61] systems generate H_2_C═C═O; (ii) the Ta_2_O_2_^+^/CH_4_/CO_2_ system [62] produces HC≡CH; and (iii) the [M(CN)]^+^/CO_2_ (M = Co, Fe) systems yield [NCCOO]^−^ [63]. These reactions demonstrate effective C−C coupling under gas‐phase conditions. We are unaware of any gas‐phase studies that have reported forming C_3_ products.

Lanthanide metals are promising alternative active sites for CO_2_ reduction. However, the mechanistic understanding of CO_2_ activation mediated by gas‐phase lanthanide‐containing ions is limited [44, 64]. Recent studies, including our work on LaB_3,4_O_2_^−^ clusters (yielding C_1_ products like CB_2_O_2_^−^, CB_3_O_3_^−^, and CB_3_O_2_^−^) [58] and the discovery of *σ* and *π* double aromaticity in PrB_2_^−^ via photoelectron spectroscopy and quantum chemical calculations [31], indicate the potential of lanthanide‐boron systems. The presence of low oxidation states of Pr in PrB_3_^−^ (+II) and PrB_4_^−^ (+I) [65] also merits further investigation as does the effect of lanthanide contraction [66] on CO_2_ reduction. These aspects motivate the targeted synthesis of lanthanide clusters with reactivity toward CO_2_ in order to generate C_3_ or C_3+_ products.

In this work, we report that the doubly aromatic PrB_2_C_2_^−^ cluster serves as an effective mediator for CO_2_ reduction, enabling the first generation of a linear C_3_B_2_O_2_^−^ species. A complete theoretical catalytic cycle is achievable through regeneration of the PrB_2_C_2_^−^ cluster via the reaction of Pr^−^ with B_2_C_2_. Related boron carbide compounds have previously been synthesized by femtosecond pulsed laser deposition, ion beam deposition, and laser chemical vapor deposition methods [67, 68, 69, 70].

## 2 Results and Discussions

### 2.1 Cluster Reactivity

**FIGURE 1:**
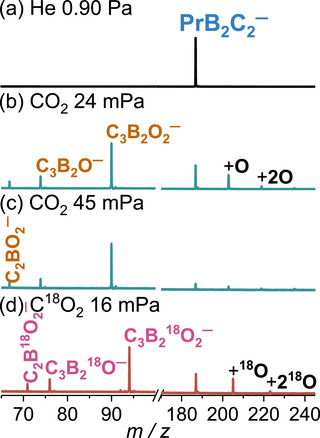
Time‐of‐flight mass spectra for the reactions of the mass‐selected PrB_2_C_2_^−^ with (a) He, (b and c) CO_2_, and (d) C^18^O_2_. The reaction time is about 0.6 ms, and the effective reactant gas pressures are shown.

As shown in Figure 1a and b, the laser‐ablation‐generated PrB_2_C_2_^−^ clusters were mass‐selected and then reacted with CO_2_ (10% in a bath of He). Three distinct peaks were observed and were assigned to C_3_B_2_O_2_^−^ (Reaction 1a), C_3_B_2_O^−^ (Reaction 1b), and C_2_BO_2_^−^ (Reaction 1c); among them, C_3_B_2_O_2_^−^ is the major product. Additionally, the PrB_2_C_2_^−^ cluster is capable of consecutively reducing two CO_2_ molecules to two CO molecules (Reaction 2, Figure 1b and c), leading to the formation of PrB_2_C_2_O_1‐2_^−^ anions.

(1a) $$\mathrm{Pr}B_{2}{C_{2}}^{-}+CO_{2}\to C_{3}B_{2}{O_{2}}^{-}+\mathrm{Pr}\;\;\;66.7\%$$

(1b) $$\begin{array}{c} \to C_{3}B_{2}O^{-}+\mathrm{PrO}\;\;23.7\% \end{array}$$

(1c) $$\to C_{2}B{O_{2}}^{-}+\mathrm{PrBC}\;\;9.6\%$$

(2) $$\mathrm{Pr}B_{2}{C_{2}}^{-}+nCO_{2}\to \mathrm{Pr}B_{2}C_{2}{O_{n}}^{-}+n\mathrm{CO}\;\left(n=1-2\right)$$

The above reaction channels were verified by an isotope labeling experiment with C^18^O_2_ (20% in a bath of He, Figure 1d). The dependence of the product ion signal on CO_2_ pressures was analyzed by pseudo‐first‐order kinetics and was fitted to the experimental data as shown in Figure S1. The CO‐generating reactions are accounted for in the peak intensity of PrB_2_C_2_^−^. The bimolecular rate constant (*k*_1_) of reaction 1 is measured to be (2.1 ± 0.4) × 10^−10^ cm^3^ molecule^−1^ s^−1^ (using equipment and equations described elsewhere [45, 71]); this corresponds to a reaction efficiency (*Φ*, defined as the ratio to the Langevin rate constant [72, 73]) of 31%. We also investigated the reactivity of PrC_2_^−^ toward CO_2_, and the products are PrC_2_O_1‐2_^−^ and CO (Figure S2), with no formation of C_3_‐backbone products. This illustrates the distinct reactivity of PrB_2_C_2_^−^.

### 2.2 Structure of PrB_2_C_2_^−^

**FIGURE 2:**
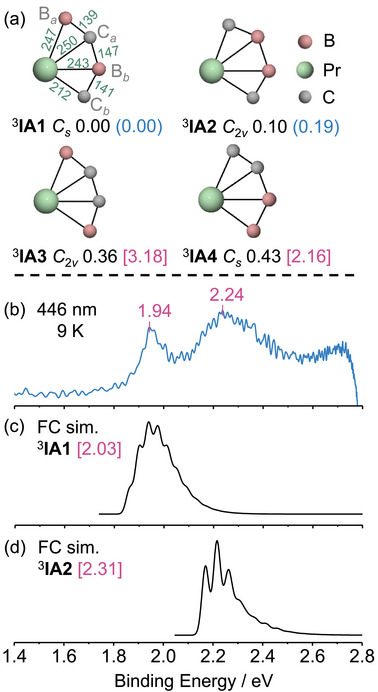
(a) PBE0/D3 structures and PBE0/D3 and QD‐SC‐NEVPT2(8,8) energies (the latter in blue parentheses) of PrB_2_C_2_^−^ structures. The bond lengths are in pm; energies are in eV; superscripts are spin multiplicities. (b) Experimental photoelectron spectrum of PrB_2_C_2_^−^ at 446 nm (2.78 eV photon energy). The ions were cooled to 9 K in an ion trap. (c and d) Simulated Franck–Condon (FC) spectra for ^3^ **IA1** and ^3^ **IA2** states of PrB_2_C_2_^−^. The calculated vertical detachment energies (VDEs in eV) are in magenta brackets; the FC spectra are red‐shifted by 0.09 eV to align better with the experiment.

The low‐lying isomers of PrB_2_C_2_^−^ that we found by DFT calculations are shown in Figures 2a and S3; these lowest‐energy isomers are triplets. The ground state structure (^3^ **IA1** in Figure 2a) adopts a planar fan‐shaped geometry with B─C─B─C bonding. Isomer ^3^ **IA2** features C─B─B─C bonds and lies 0.1 eV higher in energy than ^3^IA1. Isomers ^3^ **IA3** and ^3^ **IA4**, which contain C─C bonds, are 0.36 and 0.43 eV less stable than ^3^ **IA1**, respectively.

We also elucidated the geometric and electronic structure of PrB_2_C_2_^−^ by photoelectron spectroscopy (PES) experiments. At 446 nm laser excitation, the experimental PES spectrum (Figure 2b) exhibits a narrow band at 1.94 eV and a broader band at 2.25 eV. These features align well with the Franck–Condon simulations for isomers ^3^ **IA1** (Figure 2c) and ^3^ **IA2** (Figure 2d), both in their spectral profiles and in their vertical detachment energies (VDEs). In contrast, the larger VDEs of ^3^ **IA3** and ^3^ **IA4** suggest their absence in the cluster source, as further supported by the Franck–Condon simulations presented in Figure S4. We performed electronic structure calculations using QD‐SC‐NEVPT2(8,8) to provide additional evidence about which structure has a lower energy; this confirms that ^3^ **IA2** is 0.19 eV higher in energy than ^3^ **IA1**. These theoretical and experimental results indicate that ^3^ **IA1** is the ground state of PrB_2_C_2_^−^, with ^3^ **IA2** coexisting as a metastable isomer.

### 2.3 Reaction Mechanisms

**FIGURE 3:**
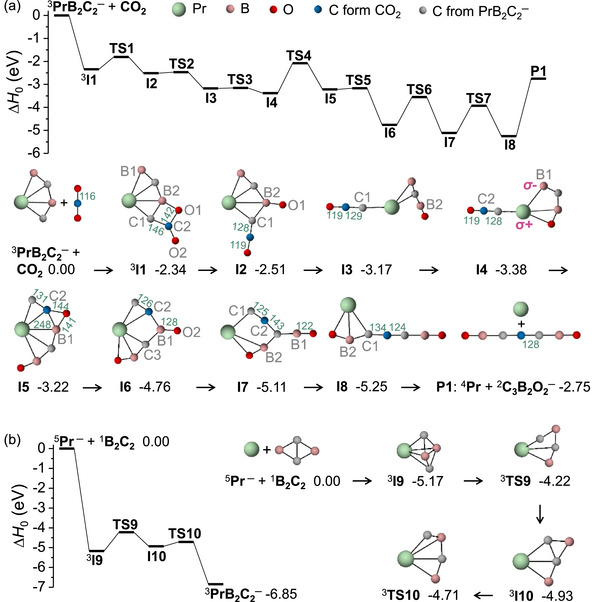
Enthalpy profiles for (a) Reaction 1a and (b) Pr^−^ with the B_2_C_2_, as calculated at the PBE0/D3 level. The relative enthalpies at 0 K (Δ*H*_0_ in eV) of reaction intermediates and products are given relative to reactants. The enthalpy at 0 K equals the sum of the potential energy and the zero‐point vibrational energy. The bond lengths are given in pm. The superscripts indicate the spin multiplicities; the system undergoes a transition from the quintet to the triplet (see Figure S10), and the reaction proceeds on a triplet potential energy surface.

The reaction mechanism of PrB_2_C_2_^−^/CO_2_ was investigated using DFT calculations. The lowest‐energy structures of C_2_BO_2_^−^, C_3_B_2_O^−^, and C_3_B_2_O_2_^−^ anions and their respective isomers are shown in Figure S5; their structural characteristics are discussed below. The most favorable reaction paths of Reactions 1a–c evolve on the triplet surfaces (Figures 3 and S6, S7), with the corresponding singlet energies provided in Table S1.

For Reaction 1a (Figures 3a and S6), the pathway initiates with CO_2_ adsorption on PrB_2_C_2_^−^ (^3^ **IA1**) to form the complex **I1**, which contains a B−C−C−O ring and represents the first C−C coupling step. This step activates the CO_2_ molecule, as evidenced by a decreased O─C─O bond angle (123°) and elongated C−O1 (142 pm) and C─O2 (121 pm) bond lengths. Subsequent C2─O1 bond cleavage via **TS1** leads to **I2**. Structural rearrangement through B2─C1 bond cleavage generates the end‐on ketenylidene (*η* ^1^‐C*_p_* C_2_O*_t_*, with the C*_p_* atom being C1 from PrB_2_C_2_^−^ and the terminal O*_t_* being O2 from CO_2_) in **I3** and **I4**. In the **I4** → **I5** step, the B1─C2 bond is formed while the O*_t_* atom forms a bridge (O*_b_* ≡O2) across C2 and B1 atoms in **I5**, forming the side‐on *η* ^2^‐C*_p_* C_2_O*_b_* ligand on Pr. Next, the Pr─B1 bond elongates from 248 pm in **I5** to 310 pm in **I6**, and the second C─O bond (which is C2─O2) originating from the CO_2_ molecule is cleaved. Subsequently, the cleavage of the B1─C2 bond (**I6** → **I7**) achieves the second C−C coupling process, forming the C_3_ backbone. Finally, the reformation of the B2─C1 bond forms a [O1─B2─C1─C2─C3─B1─O2] unit in **I8**, leading to the generation of **P1** (^4^Pr and ^2^C_3_B_2_O_2_^−^). As discuss below, PrB_2_C_2_^−^ is aromatic, and therefore the overall reaction is a dearomatization process, which is inherently energetically unfavorable but is overcome by the concomitant formation of stable chemical units (e.g., CCO, BO, and C_3_) within key intermediates.

Because the cluster source may also produce a minor amount of ^3^IA2 isomer, we also investigated its reaction pathway with CO_2_ (Figure S8). This pathway also culminates in the formation of the C_3_B_2_O_2_^−^ product. Additionally, Figure S9 presents alternative pathways for ^3^IA1 + CO_2_; however, these are less favorable than the pathway in Figure 2a because they have higher energy barriers.

In contrast to Reaction 1a, Reactions 1b and 1c lead to the formation of the C_3_B_2_O^−^ and C_2_BO_2_^−^ anions, both of which contain C_2_ units. The corresponding reaction pathways are given in Figure s7. Note that there is no C─C bond in PrB_2_C_2_^−^; thus, the C_2_ units are formed during the reactions. With respect to the separated reactants, the reactions are exothermic by −2.75 eV for **P1** (^4^Pr and ^2^C_3_B_2_O_2_^−^), −1.96 eV for **P2** (^4^PrO and ^2^C_3_B_2_O^−^), and −0.56 eV for P3 (^3^PrBC and ^1^C_2_BO_2_^−^). These computational results are consistent with experimental observations. It should be noted that Reaction 3 does not yield C_3_B_2_O_2_^−^, as this process is endothermic (Δ*H*_0_ = +2.07 eV).

(3) $$C_{3}B_{2}O^{-}+CO_{2}\to C_{3}B_{2}{O_{2}}^{-}+\mathrm{CO}$$

### 2.4 Recovery of PrB_2_C_2_^−^

**SCHEME 1:**
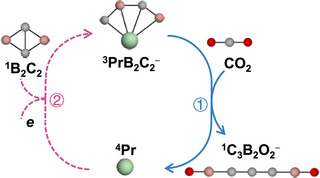
Proposed a catalytic cycle for CO_2_ activation mediated with PrB_2_C_2_^−^ anions.

We also investigated the regeneration of PrB_2_C_2_^−^ from the ^4^Pr atom (the product in Reaction 1a) via B_2_C_2_, as illustrated in Figures 3b and S10. Boron carbide compounds B_2_C_2_ have been successfully synthesized and reported; for instance, B_2_C_2_ thin films were deposited on silicon substrates via hot filament chemical vapor deposition using a precursor solution of B_2_O_3_ in methanol [74]. We used a bonding‐restricted structure search method based on the particle swarm optimization to identify a stable two‐dimensional B_2_C_2_ structure [75]. The ground state of the B_2_C_2_ molecule is of D_2*h*_ symmetry [76]. In the recovery process, PrB_2_C_2_^−^ formation begins with ^4^Pr → ^5^Pr^−^ via electron uptake. Subsequently, ^5^Pr^−^ and B_2_C_2_ undergo an exothermic reaction with an enthalpy of reaction of −5.17 eV. As shown in Figure 3b, the B─C bond cleaves in **I10** via the transition state **TS9**, followed by C─C bond breaking in **I10** over a small barrier **TS10**, leading to the regeneration of PrB_2_C_2_^−^. DFT calculations confirm that the recovery of PrB_2_C_2_^−^ is thermodynamically highly favorable, thereby successfully closing the catalytic cycle proposed in Scheme 1.

### 2.5 Orbitals and Bond Orders of PrB_2_C_2_^−^

**FIGURE 4:**
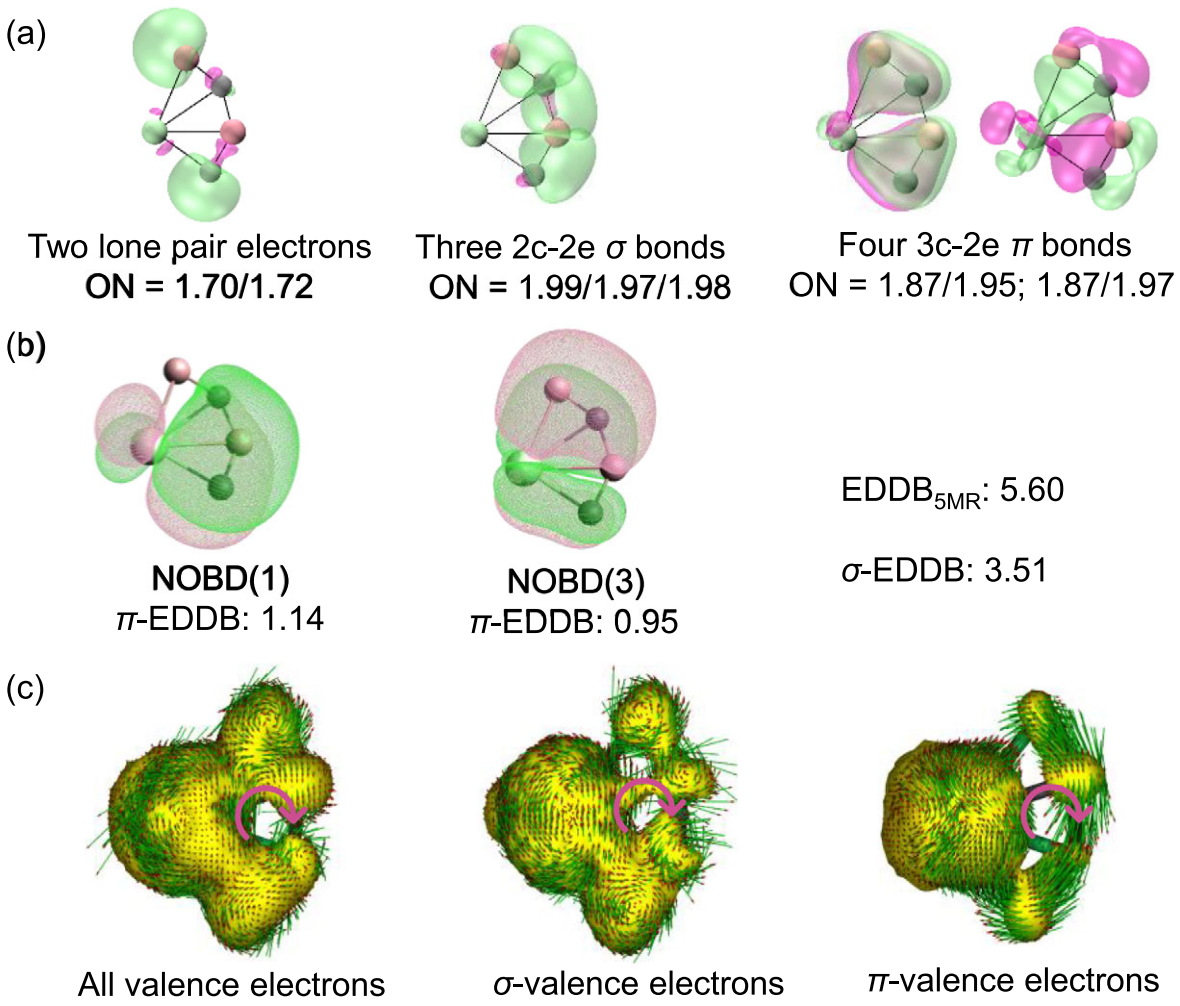
(a) Adaptive natural density partitioning bonding analysis (isovalue = 0.06) of PrB_2_C_2_^−^. ON denotes occupation number. (b) EDDB analysis (isovalue = 0.02) of PrB_2_C_2_^−^. NOBD(*n*) is natural orbital *n* of electrons in a delocalized bond. EDDB is the electron density delocalized in a bond. 5MR denotes the five‐membered ring, and 1.14 + 0.95 + 3.51 = 5.60. (c) AICD isosurfaces (isovalue = 0.05) of PrB_2_C_2_^−^ generated from all valence electrons and from the delocalized *σ* and *π* valence electrons. The magnetic field is pointing out of the molecule plane.

The AdNDP analysis reveals that ground‐state ^3^ **IA1** has nine nominally doubly occupied orbitals (shown in Figure 4a), accounting for 17.02 electrons in three two‐center–two‐electron (denoted as 2c–2e) B─C *σ* bonds, four three‐center–two‐electron (3c–2e) Pr─B─C *π* bonds, and two lone pairs (1c–2e) on the B and C atoms. There are also two singly occupied orbitals, making a triplet, and the 20th electron is distributed over many orbitals. The ^3^ **IA1** structure lacks a C─C bond; the Pr atom is connected to three alternating B─C bonds, with bond orders of 2.11, 1.36, and 1.90, respectively (the crystal structure of PrB_2_C_2_ [77], also features boron atoms alternately bonded to carbon atoms). The PrB_2_C_2_^−^ anion has a Pr─C_*a*_ single bond with bond order of 0.81 and a Pr = C_*b*_ double bond with a bond order of 1.94, while the Pr─B_*a*_ and Pr─B_*b*_ single bonds have bond orders of 1.15 and 0.95, respectively.

### 2.6 Double Aromaticity of PrB_2_C_2_^−^

The EDDB method provides a quantitative measure of electron delocalization in the conjugated system, and high delocalization is one requirement for aromaticity [78]. As shown in Figure 4b, the global EDDB value for the five‐membered ring [Pr−C−B−C−B] is 5.60 delocalized electrons, which is the sum of 2.09 delocalized *π* electrons and 3.51 delocalized *σ* electrons. This arises from contributions of 1.68 and 1.40 by the two carbon atoms, 0.82 and 0.90 by the two boron atoms, and 0.80 by the Pr atom. The *π*‐EDDB population of 2.09 satisfies the Hückel rule (4*n* + 2 with *n* = 0) and confirms *π* aromaticity. The significant *σ*‐EDDB value of 3.51 supports the presence of *σ* aromaticity, whose presence is further examined bin the next paragraph by the height dependence of the NICS value. The *σ* and *π* delocalization of PrB_2_C_2_^−^ is comparable to that of the six‐membered ring Y_2_BON_2_^+^ cation reported previously [79] to have moderate double aromaticity with a *σ*‐EDDB of 3.07 and a *π*‐EDDB of 2.46.

The NICS(*h*)*_zz_* index measured at a height *h* above the molecular plane is a measure of a diatropic anisotropy and is widely employed as an indicator of aromaticity [80]. We calculated a NICS(1 Å)_*zz*_ value of −49.7 ppm for PrB_2_C_2_^−^, which is greater than the value of −39.9 ppm for *σ*‐aromatic B_3_^−^ [31]. Furthermore, the NICS(*h*)_*zz*_ value decreases markedly with increasing height *h* above the molecular plane (−213.6, −153.4, and −49.7 ppm for *h* equal to 0, 0.5, and 1.0 Å, respectively). This sharp attenuation is characteristic of systems with dominant *σ*‐aromaticity, because the effect of the in‐plane *σ* bonds decays rapidly away from the ring center. This behavior is similar to that observed in the doubly aromatic PrB_2_^−^ anion (−284.6, 172.0, and −62.5 ppm for the same values of *h*) [31].

Furthermore, the AICD isosurfaces [81] of PrB_2_C_2_^−^ (Figure 4c) reveal a diatropic current at the geometric center for the all‐valence electrons, for the *σ* valence electrons, and for the *π* valence electrons, confirming the double aromaticity.

In summary, the EDDB analysis, the NICS, and the AICD are all consistent with the double 4*f*‐metalla‐aromaticity in the PrB_2_C_2_^−^ anion.

### 2.7 Structural Characteristics of the New Species C*_x_* B*_y_* O*_z_* ^−^ (*x* = 2 or 3, *y* = 1 or 2, *z* = 1 or 2)

**FIGURE 5:**
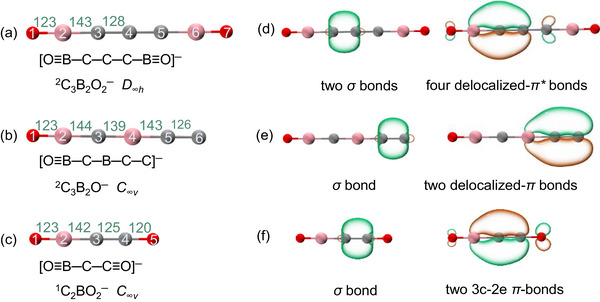
Optimized geometries with key bond lengths (in pm) and their valence bond representations for (a) C_3_B_2_O_2_^−^, (b) C_3_B_2_O^−^, and (c) C_2_BO_2_^−^ anions. Natural localized molecular orbitals predominantly responsible for the central C─C bonds in (d) C_3_B_2_O_2_^−^, (e) C_3_B_2_O^−^, and (f) C_2_BO_2_^−^.

The anions C_3_B_2_O_2_^−^, C_3_B_2_O^−^, and C_2_BO_2_^−^ are produced in Reactions 1a–c. Their optimized geometries and valence bond structures are shown in Figure 5. All three species adopt linear structures built from B≡O triple bonds and B─C bonds [58]. The previous work has established boronyl (BO) as a monovalent *σ* radical [82]. The calculated B─C bond length in C_3_B_2_O_2_^−^ (143 pm) agrees well with the typical C(sp^2^)─B bond length of 144 pm [83], suggesting bond orders of about two. Natural localized molecular orbital analysis (Figures 5d–f and S11–S13) reveals the presence of delocalized C─C bonds in these systems. The corresponding C─C bond lengths are 128 pm (in C_3_B_2_O_2_^−^), 126 pm (in C_3_B_2_O^−^), and 126 pm (in C_2_BO_2_^−^), which fall between standard values for a C═C double bond (134 pm) and a C≡C triple bond (120 pm).

As illustrated in Figure 5a, the C_3_B_2_O_2_^−^ anion contains a C−C−C unit. Each C─C bond includes a *σ*‐bond, while four delocalized‐*π* bonds span the B2−C3−C4 (and symmetrically equivalent C4−C5−B6) unit, with additional π*‐type interactions involving the C5 (or C3) atom (Figure 5d). In contrast, both C_3_B_2_O^−^ and C_2_BO_2_^−^ contain only one C─C bond (Figure 5b,c). In C_3_B_2_O^−^, the B4−C5−C6 unit features one *σ*‐bond between the carbon atoms, along with two delocalized‐*π* bonds (Figure 5e). A lone pair of electrons resides on the terminal C6 atom. Meanwhile, the C_2_BO_2_^−^ anion exhibits a B2−C3−C4 unit with one *σ*‐bond and two three‐center–two‐electron *π*‐bonds (Figure 5f).

### 2.8 Key Factors of C_3_ Unit Formation

**SCHEME 2:**
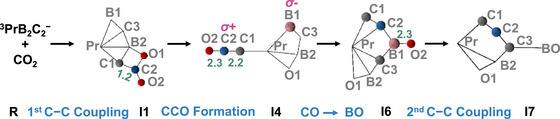
Skeletal reaction mechanism for the formation of the C_3_ unit from the reaction of PrB_2_C_2_^−^ with CO_2_. The Wiberg bond index values are given in green.

Mass spectrometry and DFT calculations confirm the formation of the C_3_‐containing product in Reaction 1a. The skeletal reaction mechanism in Scheme 2 comprises three major stages featuring two critical C−C coupling steps.

**FIGURE 6:**
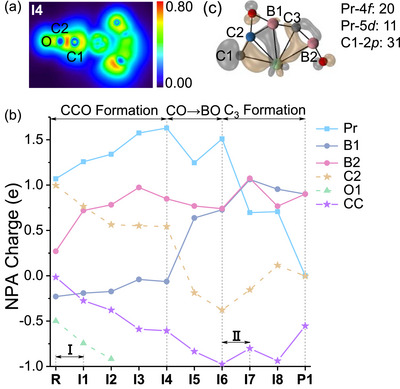
(a) Localized orbital locators (unitless) of intermediate **I4**. (b) Natural population analysis charges along the reaction coordinates of Reaction 1a. The CC unit (purple stars) consists of the C1 and C2 atoms. Steps **I** and **II** correspond to the two C─C bond coupling processes. The processes of CCO formation, conversion of terminal CO to BO, and C_3_ formation are also marked. (c) The highest occupied molecular orbital of intermediate **I5**. The isosurface value is set to 0.05 a.u. The leading contributors to the orbital composition as obtained by the natural atomic orbital method are shown as percentages.

In the first stage, two‐dimensional localized orbital locator analysis indicates the transformation from **I2** to **I4** yields an end‐on coordinated CCO group (*η* ^1^‐CCO) forming in the initial C−C coupling step where electrophilic CO_2_ attacks the aromatic PrB_2_C_2_^−^ (**R** → **I1**), with the Pr^III^ center uniquely mediating bond cleavage and bond formation processes involving Pr─B, Pr─C, and Pr─O bonds. A higher value of the localized orbital locator indicates a more localized electron distribution as in a covalent bond, and the analysis in Figure 6a reveals significant electron accumulation in C─C─O bonding regions, where we find bond orders of ∼2.0 for C1−C2 and ∼2.3 for C2−O2 in **I2** to **I4**.

In the second stage, the terminal CO converts to BO. This transformation process is propelled by two principal factors: (1) the electrostatic attraction between the negatively charged B1*^δ^* ^−^ and the positively charged C2*^δ^* ^+^ atoms in **I4**, which drives B1─C2 bond formation in **I6**; and (2) the higher bond dissociation enthalpy of B−O (806 kJ/mol) compared to C−O (728 kJ/mol) within the CCO unit, which enhances thermodynamic stability and effectively suppresses an alternative CO‐producing pathway.

The final stage involves the second C−C coupling event, culminating in the assembly of the complete C−C−C skeleton and yielding the product identified by mass spectrometry. Therefore, the aromaticity of PrB_2_C_2_^−^ not only facilitates the initial CO_2_ adsorption but also, through the release of ring strain upon dearomatization, drives the subsequent steps, including C─CO bond formation, CCO unit generation, and the ultimate C−CC coupling.

### 2.9 Roles of Pr and B Atoms

We further elucidated the pivotal roles of Pr and B atoms across the three major reaction regions through natural population analysis (natural population analysis, Figure 6b). For the sake of clarity, the charges of C1 and O2 are omitted due to their variation being small during the reaction. During CCO formation (**R** → **I4**), Pr and two B atoms collectively function as electron donors (Δ*Q* = 1.30 e), facilitating CO_2_ activation and enabling the first C−C coupling. Subsequently, the Pr atom acts as an electron shuttle, converting terminal CO to BO (**I4** → **I6**). Notably, B1 behaves as a Lewis base in this process, donating 0.79 e to form a bond with C2. While boron is conventionally considered electron‐deficient and typically exhibits Lewis acidic property [82], its nucleophilic character here is modulated by the electropositive nature of Pr. In this system, the higher electronegativity of B (2.04) compared to Pr (1.13) polarizes the Pr*^δ^* ^+^─B*^δ^* ^−^ bond, thereby conferring nucleophilicity to the B1 atom. For the C_3_ formation step (**I6** → **I7**), B1, B2, and C2 collectively serve as electron donors (donating a total of 0.89 e), while Pr functions as an electron acceptor, facilitating the second C−C coupling. The essential roles of Pr and B atoms are summarized as follows: (1) The Pr^III^ center demonstrates pronounced chemical versatility, enabling dynamic formation and cleavage of Pr−*X* (*X* = B, C, O) bonds. (2) The nucleophilic character of B establishes a precondition for the conversion of CO to BO, kinetically suppressing the competitive CO release pathway. (3) These centers function synergistically as electron shuttles, facilitating C_3_ skeleton assembly.

**SCHEME 3:**
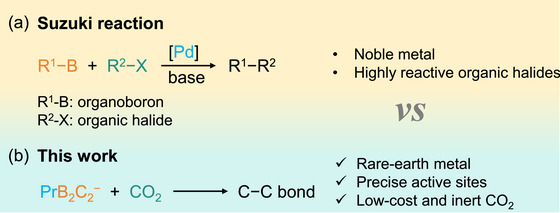
Comparison of C−C coupling strategies: (a) conventional Suzuki cross‐coupling vs. (b) the approach reported in this work.

The fundamental importance of C─C bond formation is highlighted by the 2010 Nobel Prize in Chemistry, awarded for pioneering Pd‐catalyzed cross‐couplings via the Heck, Negishi, and Suzuki reactions. Scheme 3a compares the novel strategy for C─C bond formation and carbon chain elongation of the present work to the conventional Suzuki cross‐coupling reaction. The Suzuki reaction requires a noble Pd‐based catalyst, a base, an organoboron species, and an expensive and highly reactive organic halide [84]. In contrast to the reliance on organic halides, our gas‐phase approach (Scheme 3b) employs low‐cost, inert CO_2_ as a coupling partner, enabled by a PrB_2_C_2_^−^ cluster that features B─C bonds, precise active sites, and a rare‐earth Pr center. The mechanism proposed in this work bears an analogy to the classic Suzuki coupling: both involve coupling a metal─carbon bond (herein, the Pr─C bond) to an electrophile (herein, CO_2_), with boron playing a key role.

In the condensed phase, the ketenylidene unit has been proposed as a significant intermediate in Fischer–Tropsch synthesis [85, 86], analogous to the *η* ^1^‐C_*p*_CO*_t_* in the PrB_2_C_2_^−^/CO_2_ system. For instance, CO_2_ adsorbs on the C*_s_* sites of the Mo_12_C_12_/Au(111) surface, forming C_*s*_═CO_2_ bonds and then converting to the C*_s_* CO unit, during the catalytic reaction [86]. In comparable gas‐phase systems such as Nb_3_C_4_O^−^/CO_2_ and RhTaC_2_O_3_^−^/CO_2_, the CCO*_t_* unit tends to release CO readily due to the stability of the C≡O*_t_* triplet bond (Figure S14) [51, 87]. Note that in the Nb_3_C_4_O^−^ cluster, the C atom of the *η* ^1^‐CCO*_t_* unit bridges two Nb atoms, whereas in the RhTaC_2_O_3_^−^ cluster, it coordinates with the Rh and O atoms. In contrast, the Pr system exhibits distinct behavior: higher‐lying Pr‐4*f* orbitals engage in significant overlap with the C_2*p*_ orbitals (Figure 6c, showing the unusual higher participation in bonding of 4*f* orbitals than 3*d* orbitals). The robust orbital interaction between the Pr and C1(C2) atoms, culminates in the formation of a C─O single bond. This interaction serves to prevent the cleavage of the C═C double bond and facilitates the subsequent conversion of CO to BO. Therefore, the judicious selection of Pr and B atoms provides an efficient strategy for promoting C−C coupling during CO_2_ activation, highlighting the unique capability of lanthanides–boron cooperation in controlling complex molecular rearrangements. Lanthanide metals can offer complementary or alternative selectivity rather than a simple superiority in rate. This mechanistic insight advances the fundamental understanding of lanthanide‐based catalysis.

## 3 Conclusion

We have synthesized and characterized the novel cluster PrB_2_C_2_^−^. Although lanthanides, which feature highly contracted 4*f* atomic orbitals, are rarely incorporated into multiple aromatic systems, the 4*f*‐metal system PrB_2_C_2_^−^ exhibits *σ* and *π* double aromaticity. Its reactivity with CO_2_, investigated through mass spectrometry, photoelectron imaging spectroscopy, and density functional theory calculations reveal a unique pathway for room‐temperature C−C−C coupling. The PrB_2_C_2_^−^ cluster undergoes CO_2_ adsorption and dearomatization processes, enabling sequential C─C─C bond formation and yielding a linear C_3_ backbone coordinated by two BO ligands. Theoretical analysis further establishes a complete catalytic cycle in which PrB_2_C_2_^−^ is regenerated via reaction of Pr^−^ with B_2_C_2_.

Beyond simple CO release, the system produces a family of previously unknown anionic species (C_3_B_2_O_2_^−^, C_3_B_2_O^−^, C_2_BO_2_^−^), whose bonding patterns illuminate the synergistic mechanistic roles of Pr‐mediated electron shuttling and nucleophilic boron centers to facilitate two C−C coupling steps while suppressing CO release.

This work demonstrates a selective gas‐phase route for CO_2_‐to‐C_3_ conversion and suggests the potential for constructing even larger carbon frameworks. By drawing conceptual parallels to Suzuki cross‐coupling, our results open new avenues for lanthanide‐cluster‐driven C─C bond formation and broaden the landscape of CO_2_ activation by organolanthanid complexes. The fundamental insights herein provide critical guidelines for lanthanide‐boride‐carbide catalysts in condensed‐phase applications.

## Conflicts of Interest

The authors declare no conflicts of interest.

## Supporting information



Details of experimental and theoretical methods, as well as additional results, including time‐of‐flight mass spectra, optimized structures, potential energy profiles, natural localized molecular orbitals, and *π*‐electron localized orbital locators.
**Supporting File**: anie72187‐sup‐0001‐SuppMat.pdf.

## Data Availability

The data that supports the findings of this study are available in the Supporting Information of this article.
