# Supplementary material for: Room‐Temperature Gas‐Phase CO2‐to‐C3 Coupling by a 4f‐Aromatic Cluster
Source: Angew Chem Int Ed Engl. 2026 Apr 14;65(22):e6756333. doi: 10.1002/anie.6756333 (PMC13206479; doi:10.1002/anie.6756333)
Supplement: Supplementary file 1 — Details of experimental and theoretical methods, as well as additional results, including time‐of‐flight mass spectra, optimized structures, potential energy profiles, natural localized molecular orbitals, and π‐electron localized orbital locators. Supporting File: anie72187‐sup‐0001‐SuppMat.pdf. [file ANIE-65-e6756333-s001.pdf]

SUPPORTING INFORMATION

Room-Temperature Gas-Phase CO_2-to-C_3 Coupling by a 4*f*-Aromatic Cluster

Feng-Xiang Zhang,[a] Xiao-Wang Li,[a] Ning-Zheng Li,[b] Sheng-Gui He,[b] Yu-Chen Zhang,[c] Li-Li Xing,[d] Donald G. Truhlar,[e] and Jia-Bi Ma[a]

[a] Key Laboratory of Cluster Science of Ministry of Education, Beijing Key Laboratory of Photoelectronic/Electrophotonic Conversion Materials, School of Chemistry and Chemical Engineering, Beijing Institute of Technology, Beijing 102488, China.

[b] Key Laboratory for Structural Chemistry of Unstable and Stable Species, Institute of Chemistry, Chinese Academy of Sciences, Beijing 100190, China; Beijing National Laboratory for Molecular Sciences and CAS Research/Education Center of Excellence in Molecular Sciences, Beijing 100190, China.

[c] Center of Basic Molecular Science, Department of Chemistry, Tsinghua University, Beijing, China.

[d] Energy and Power Engineering Institute, Henan University of Science and Technology, Henan 471003, China.

[e] Department of Chemistry, Chemical Theory Center, and Minnesota Supercomputing Institute, University of Minnesota, Minneapolis, Minnesota 55455-0431, United States.

***Corresponding authors.** E-mails: xingll2018@haust.edu.cn; truhlar@umn.edu; majiabi@bit.edu.cn

March 29, 2026

Contents

1. **Details of experimental and theoretical methods**
2. **Additional experimental results** (Figures S1-S2)
3. **Additional theoretical results** (Figures S3-S14; Tables S1)
4. **References**

1. Details of experimental and theoretical methods

1.1 Experimental methods

The $PrB_xC_y^-$ clusters were generated by laser ablation of a rotating and translating Pr:B target disk (molar ratio Pr:B=1:3 with monoisotopic ^{11}B) in a 5% CD4 / 95% helium (He) gas mixture with a backing pressure of 4 atm. We use a 532 nm (second harmonic of Nd^{3+}: yttrium aluminum garnet) laser with an energy of 5−8 mJ per pulse and a repetition rate of 10 Hz. The dominant $PrB_2C_2^-$ ions were mass-selected by a quadrupole mass filter and enter the linear ion trap reactor, where they were confined and thermalized by collisions with a pulse of He and then interact with a pulse of reactants (CO_2 or $C^{18}O_2$) for a period of time. It has been shown in previous studies that the ions in such an experiment are thermalized to (or close to) room temperature before reactions.[1,2] The ions ejected from the linear ion trap were detected using a reflection time-of-flight mass spectrometer. The method to derive the rate constants is described in detail in Ref 3.

Photoelectron imaging spectroscopy of the reactant cluster $PrB_2C_2^-$ was performed using a separate vacuum apparatus with three main components: a laser ablation cluster source, a linear ion trap, and a photoelectron imaging spectrometer. The details of the experimental setup have been described in Ref 4, and here we provide here only an overview of the photoelectron imaging experiments. Following the same procedure as employed in the previous experiments, the $PrB_2C_2^-$ anions were generated and cooled in a cryogenic ion trap with a temperature of 9 K. After a 90 ms trapping period, the target ions were irradiated with a 446 nm laser beam from an optical parametric oscillator laser. The kinetic energies of the photodetached electrons were measured using the photoelectron imaging spectrometer.

1.2 Theoretical methods

All electronic structure calculations employing density functional theory (DFT) were performed using the *Gaussian 09–D.01* software package.[5] We employed the PBE0/D3 exchange-correlation functional, where D3 (sometimes called D3(0)) denotes an empirical damped dispersion term.[6] (Previous work showed that PBE0 gives reasonable predictions for structures containing praseodymium atoms,[7–10]) The 6-311+G* basis set[11,12] is used for B, C, and O atoms, and the Stuttgart-type relativistic effective core potential basis with 28 core electrons (basis set ECP28MWB_ANO) was employed for the Pr atom.[13,14]

The initial search for isomers of various clusters was conducted using the *ABCluster* program.[15,16] In reaction pathways, each reaction intermediate (I) and transition state (TS) was geometrically optimized; the intermediates and transition states were verified to have respectively zero and one imaginary vibrational frequency. Paths of steepest descent were calculated to confirm that each TS connects the appropriate intermediates. The nucleus-independent chemical shift[17] (NICS), the anisotropy of the induced current density[18] (AICD), and two-dimensional localized orbital locator[19,20] analyses were obtained using *Multiwfn 3.8*.[21] The adaptive natural density partitioning (AdNDP) analysis,[22] the natural population analysis charges, natural localized molecular orbitals,[23] and bond orders (Wiberg bond indices) were calculated with the *NBO 6.0* software.[24] Based on the natural atomic orbitals calculated using

the *NBO 6.0*, the electron density of delocalized bonds[25] (EDDB) was analyzed with the *RunEDDB* script.[26]

We also carried out Franck-Condon simulations of excitation spectra at 0 K. Spectral line broadening was modeled using a Gaussian function with a full width at half-maximum (FWHM) of 135 cm^{-1}. All other parameters in these simulations were derived from DFT computations.

All calculations except for single-point energies calculated by quasi-degenerate strongly contracted *n*-electron valence state perturbation theory[27–29] (QD-SC-NEVPT2) were carried out with PBE0/D3. We calculated the energy differences of the $PrB_2C_2^-$ isomers by QD-SC-NEVPT2 using the *ORCA 6.0.1* software package.[27–31] These calculations start with state-averaged complete active space self-consistent field (CASSCF) calculations; the initial orbitals for the CASSCF iterations were natural orbitals derived from unrestricted Hartree-Fock calculations. We employed an active space of 8 electrons in 8 orbitals, [CASSCF(8,8)] with the four highest occupied natural orbitals and the four lowest unoccupied natural orbitals in the active space. The subsequent QD-SC-NEVPT2(8,8) calculations involve five singlets and five triplets; they allow for mixing of the SA-CASSCF states by quasidegenerate perturbation theory, and they incorporate additional dynamic correlation to give more reliable energy levels. The def2-SVP basis set was used for these calcualtions.[32]

2. Additional experimental results

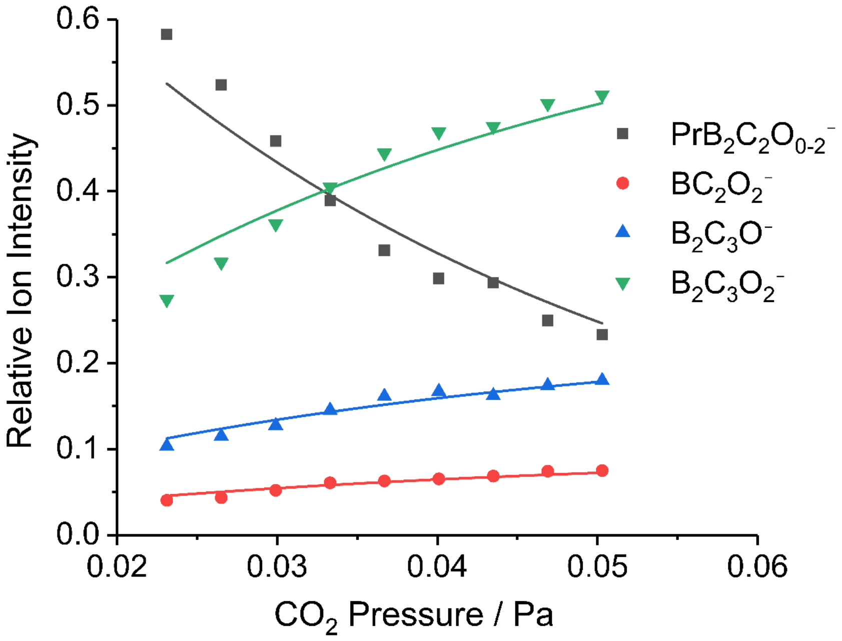

Figure S1. Variations of the relative intensities of the reactant and product anions in the reaction of $PrB_2C_2^-$ and CO_2 with respect to the CO_2 pressures for 0.6 ms. The solid lines are fitted to the experimental data points by using the equations derived with the approximation of the pseudo-first-order reaction mechanism.

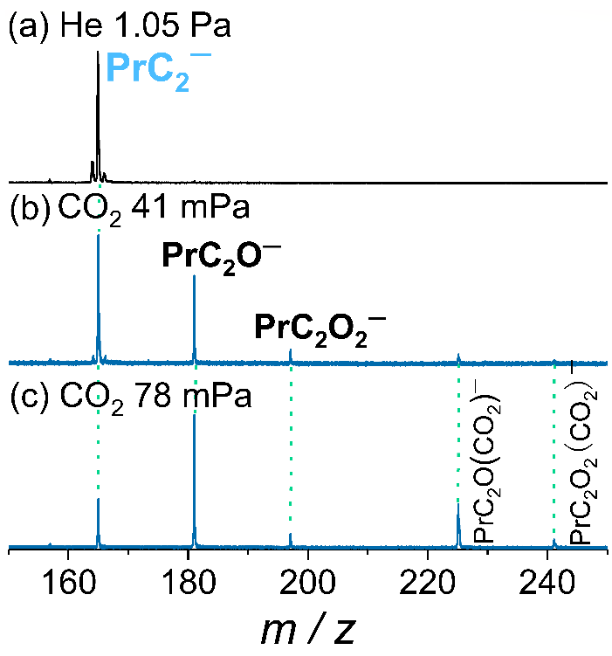

Figure S2. Time-of-flight mass spectra for the reactions of mass-selected PrC_2^- with (a) He, (b and c) CO_2. The reaction time is about 0.8 ms. The reaction was performed using a mixture of gases. The effective gas reactant pressure is shown and was calculated from the following equation:[33]

$$P \equiv P_{\max}\frac{\tau_R}{t_R}\left[1+\frac{\delta t_R}{2\tau_R}-e^{-(t_R-\delta t_R)/\tau_R}\right]$$

where $P_{\max}$ is the instantaneous gas pressure in the ion trap when the pulsed valve is closed, and τ_R and $\delta\tau_R$ are respectively the decay time and pulse width of reactant gas. Detailed information can be found on page S8 of the Supporting Information (Ref 33).

3. Additional theoretical results

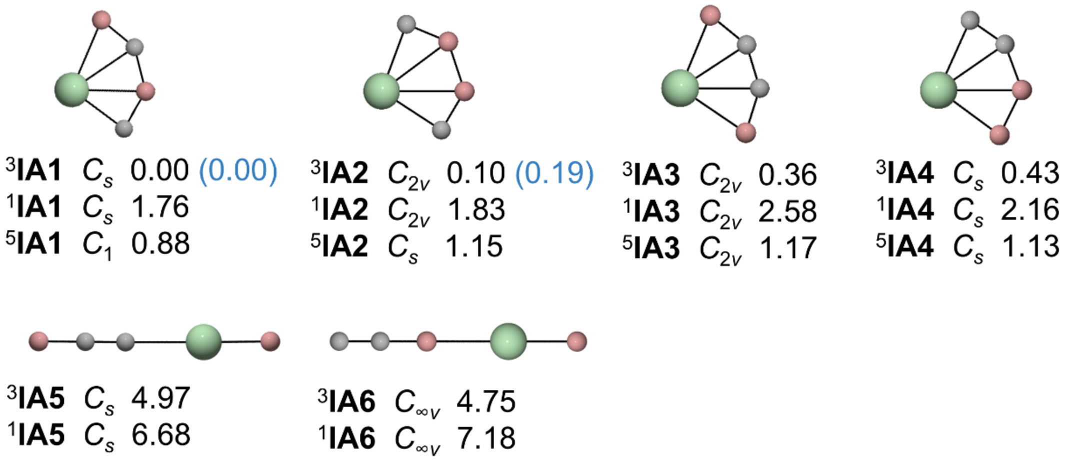

Figure S3. Structures optimized by PBE0/D3 and energies (in eV) calculated by PBE0/D3 and single-point QD-SC-NEVPT2(8,8) calculations (the latter in blue) for the lower-energy $PrB_2C_2^-$ isomers with relative lower energies. The superscripts indicate spin multiplicities.

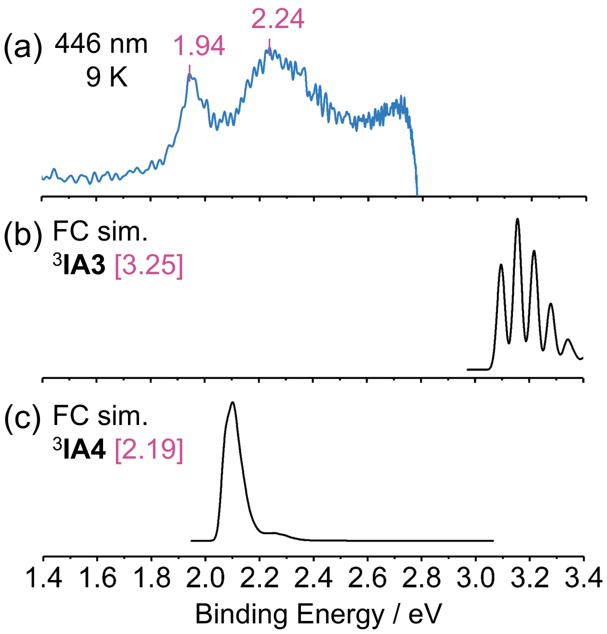

Figure S4. (a) Experimental photoelectron spectrum of $PrB_2C_2^-$ at 446 nm (2.78 eV photon energy). The ions were cooled to 9 K in an ion trap. (b and c) Simulated spectra for ^{3}**IA3** and ^{3}**IA4** states of $PrB_2C_2^-$, calculated based on the Franck-Condon principle. The calculated FC spectra are red shifted by 0.09 eV to better align the theoretical spectrum with the experimental one. The calculated vertical detachment energies (VDEs in eV)) are given in the magenta. The superscripts indicate spin multiplicities.

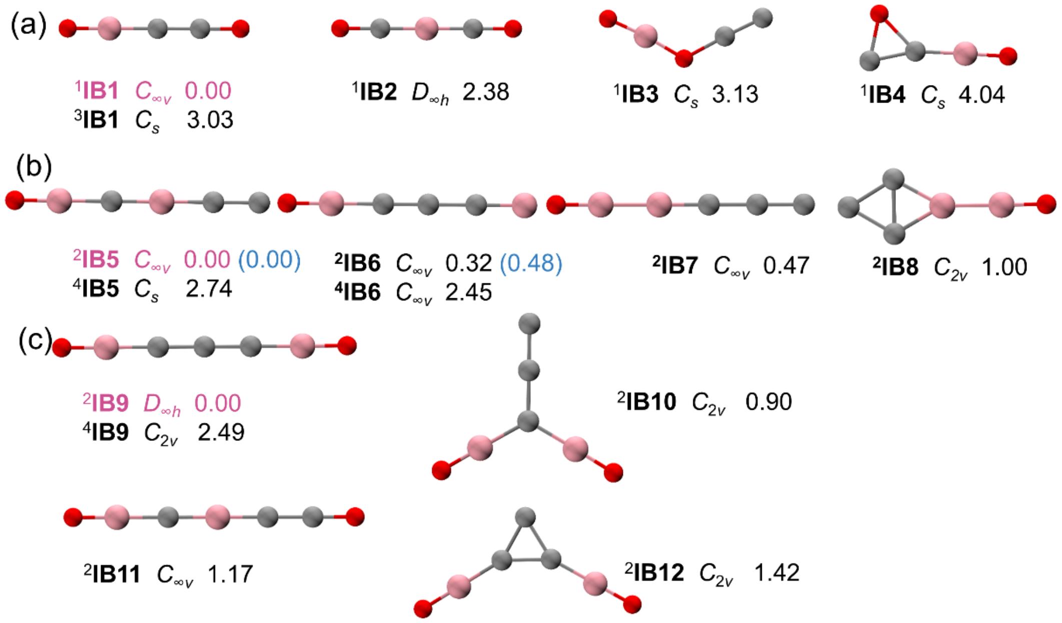

Figure S5 Structures optimized by PBE0/D3 and energies (in eV) calculated by PBE0/D3 and single-point QD-SC-NEVPT2(8,8) calculations (the latter in blue) for the low-lying isomers of (a) $C_2BO_2^-$, (b) $C_3B_2O^-$, and (c) $C_3B_2O_2^-$. The superscripts indicate spin multiplicities.

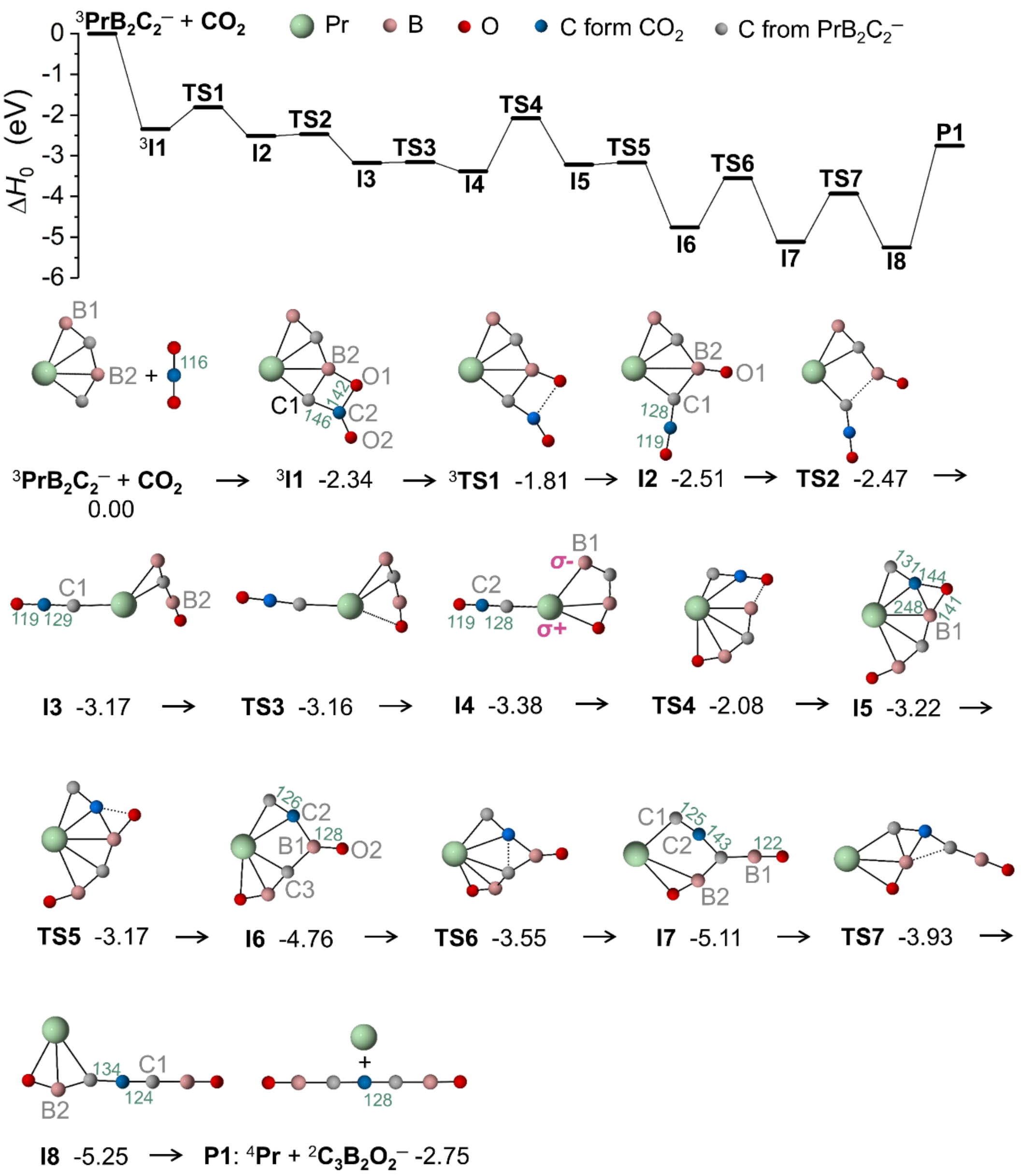

Figure S6. The enthalpy profile (ΔH_0 in eV) of reaction intermediates and products for reaction 1a, as calculated at the PBE0/D3 level. The enthalpy is given at 0 K, where it is the sum of potential energy and zero-point vibrational energy; all enthalpies are relative to the separated reactants. Selected bond lengths are given in pm. All pathways evolve along the triplet surface, and the superscripts indicate the spin multiplicities.

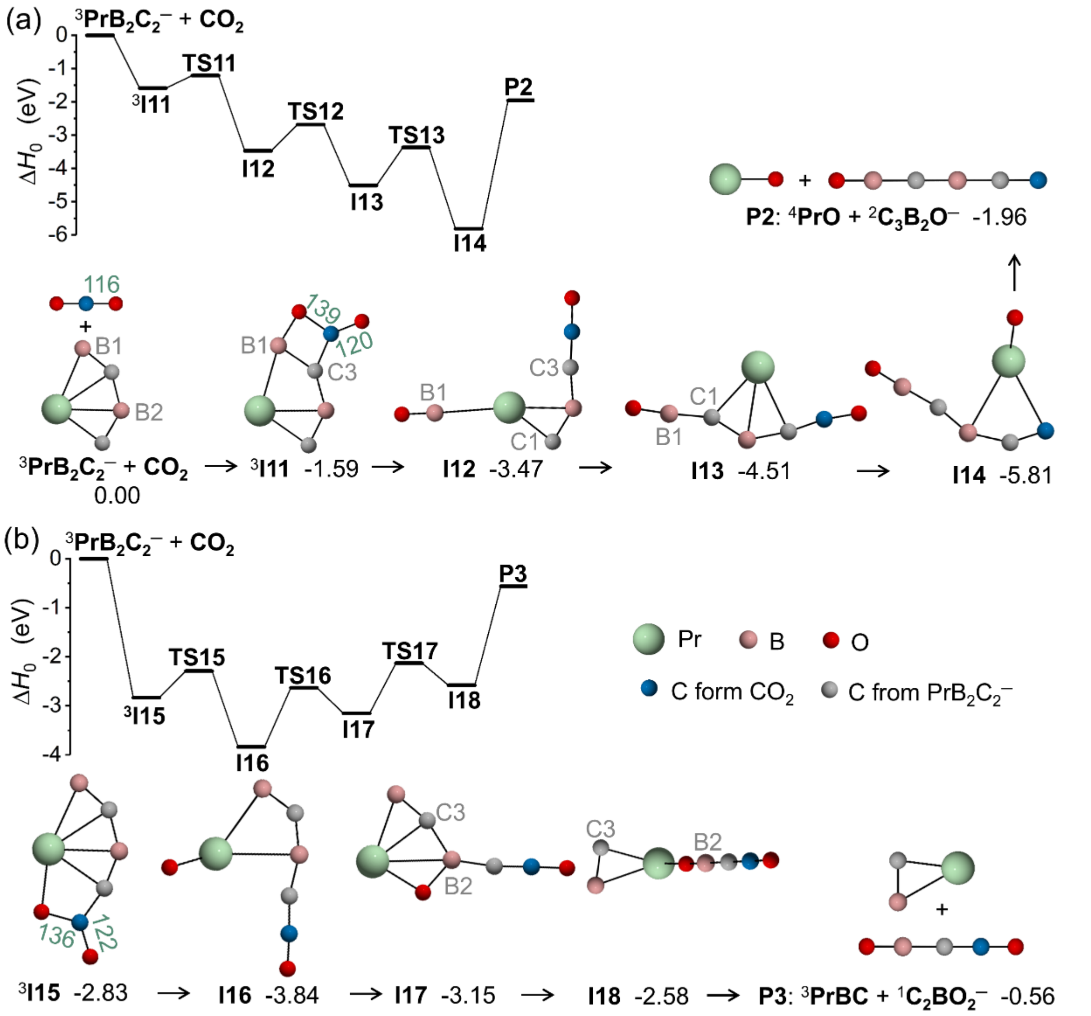

Figure S7. Enthalpy profiles (ΔH_0 in eV) of reaction intermediates and products for reactions (a) 1b and (b) 1c, as calculated at the PBE0/D3 level. All pathways evolve along the triplet surface, and the superscripts indicate the spin multiplicities; the spin multiplicities of all species whose spin multiplicity is not indicated in the figure are triplet.

The reaction pathways of $C_3B_2O_2^-$, $C_2BO_2^-$, and $C_3B_2O^-$ anions from CO_2, mediated by the $PrB_2C_2^-$ cluster, are presented in Figures 3 and S7 (Supporting Information). Although the initial adsorption configurations differ, the carbon atom of CO_2 consistently binds to the carbon sites of the $PrB_2C_2^-$ cluster in all cases. Our analysis focuses on $C_3B_2O_2^-$ as the primary product. The two alternative pathways, which originate from distinct adsorption configurations, are provided in Figure S9. Theoretical results reveal that these pathways are thermodynamically less favorable than the pathway detailed in Figure 3. Therefore, the initial adsorption configuration is a key determinant of product selectivity. Furthermore, the formation of the $C_3B_2O_2^-$ species (which incorporates a C_3 unit) requires two C−C coupling steps. In contrast, the generation of both $C_2BO_2^-$ and $C_3B_2O^-$ anions, each containing a C_2 unit, involves only a single C−C coupling step.

Table S1. Singlet relative enthalpies (in eV as calculated by PBE0/D3) along the reaction pathways for the generation of $C_3B_2O_2^-$, $C_3B_2O^-$, and $C_2BO_2^-$. The energy is set to zero for reactants ($^3PrB_2C_2^-$ + CO_2) in their ground state.

$C_3B_2O_2^-$		$C_3B_2O^-$		$C_2BO_2^-$	
**I1**	-0.57	**I11**	0.13	**I15**	-1.08
**TS1**	-0.05	**TS11**	0.53	**TS15**	-0.57
**I2**	-0.81	**I12**	-1.69	**I16**	-1.46
**TS2**	-0.74	**TS12**	-0.91	**TS16**	-1.10
**I3**	-1.39	**I13**	-2.63	**I17**	-1.32
**TS3**	-1.37	**TS13**	-1.61	**TS17**	-0.39
**I4**	-1.16	**I14**	-4.08	**I18**	-0.81
**TS4**	-0.34				
**I5**	-1.48				
**TS5**	-1.43				
**I6**	-2.50				
**TS6**	-1.70				
**I7**	-3.05				
**TS7**	-1.89				
**I8**	-3.20				

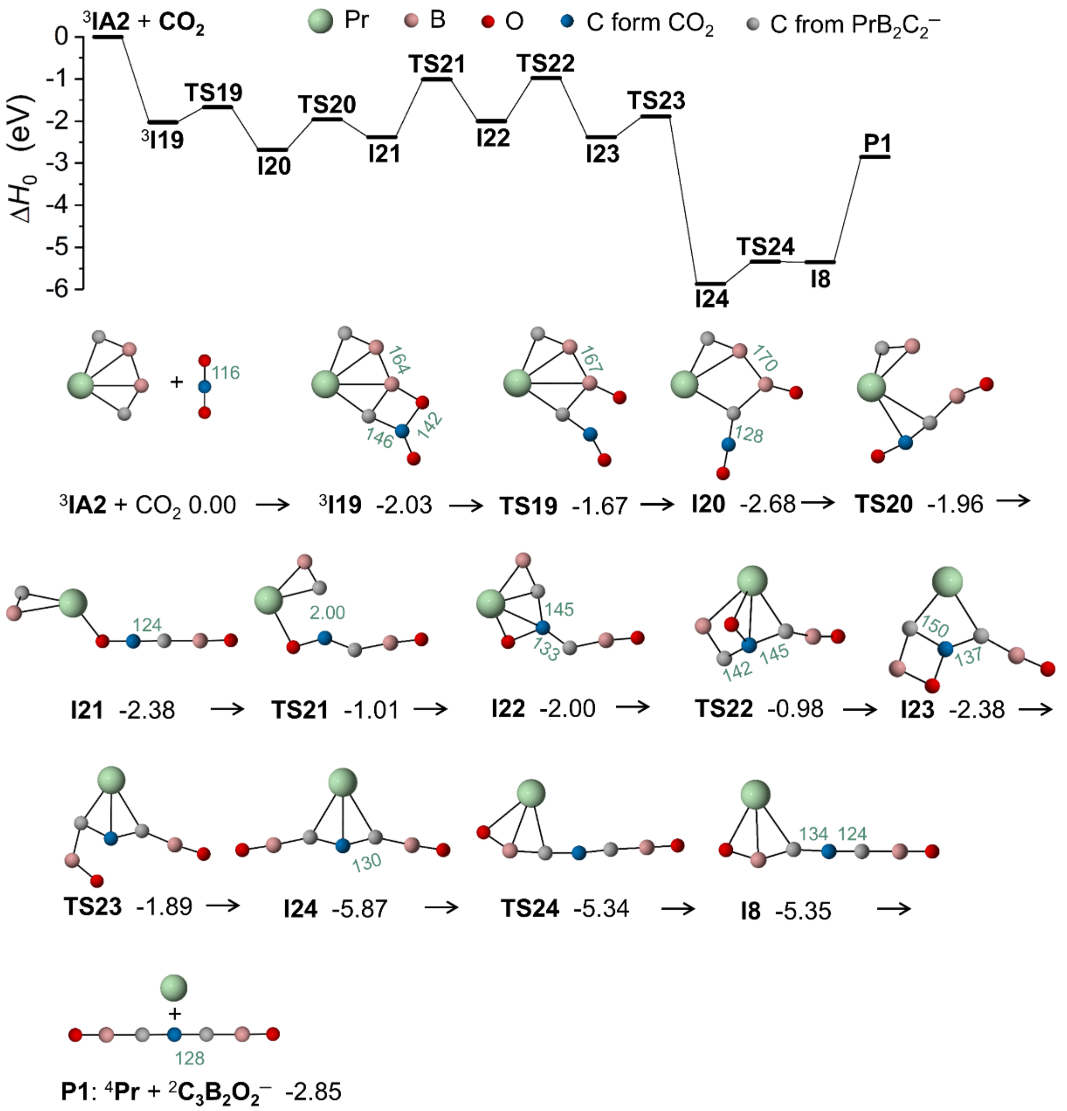

Figure S8. The enthalpy profile (ΔH_0 in eV) of reaction intermediates and products for reaction of ^{3}**IA2** and CO_2. The enthalpy is given at 0 K, where it is the sum of potential energy and zero-point vibrational energy; all enthalpies are relative to the separated reactants. Selected bond lengths are given in pm. All pathways evolve along the triplet surface, and the superscripts indicate the spin multiplicities.

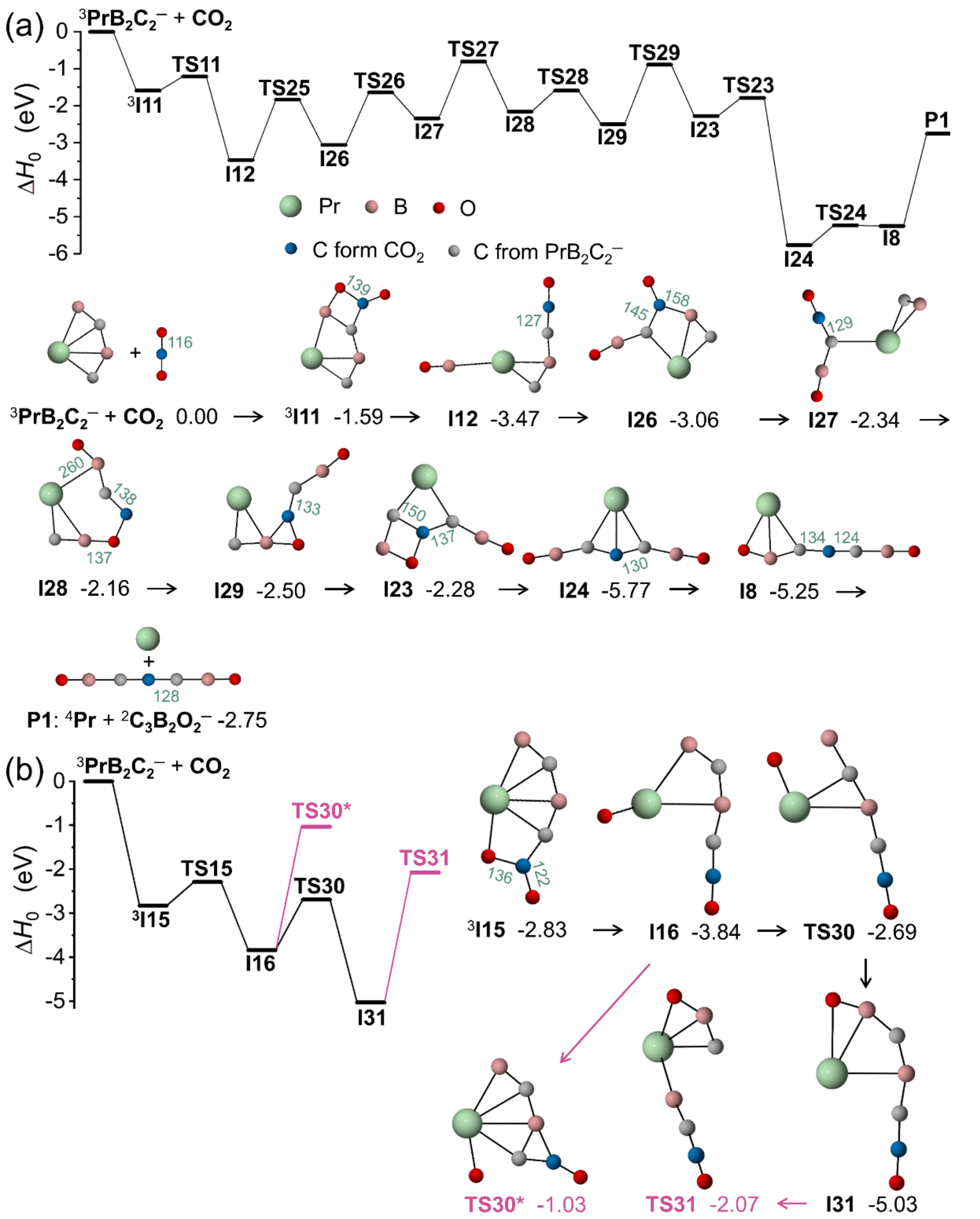

Figure S9. The enthalpy profile (ΔH_0 in eV) of reaction intermediates and products for reaction 1a, starting from the initial CO_2 adsorption structures of **I11** (a) and **I15** (b). The enthalpies are given at 0 K, where they are the sum of potential energy and zero-point vibrational energy; all enthalpies are relative to the separated reactants. Selected bond lengths are given in pm. All pathways evolve along the triplet surface, and the superscripts indicate the spin multiplicities. The reaction pathway following **TS31** is omitted in panel b due to the higher energy barrier of step **I31** → **TS31** (2.96 eV) compared to the maximum barrier (2.5 eV) identified along the favorable pathway detailed in Figure S6.

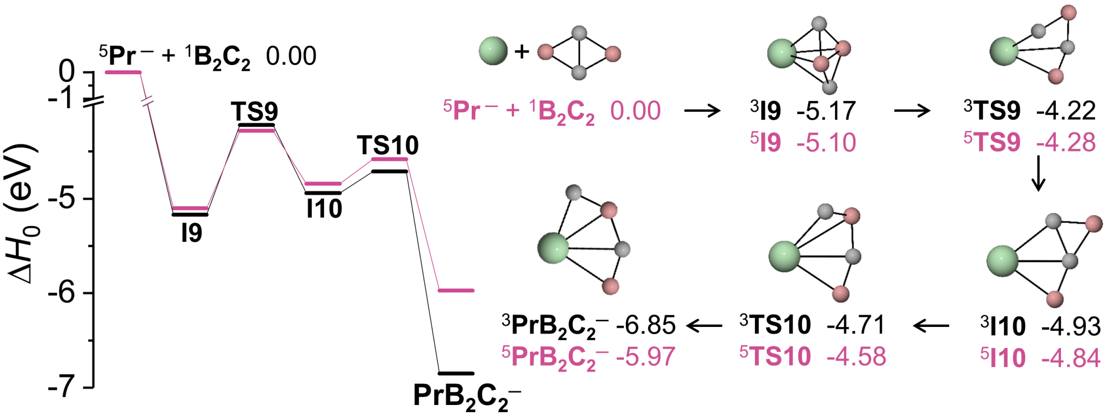

Figure S10. Enthalpy profiles for the reaction of Pr^- with B_2C_2, as calculated at the PBE0/D3 level. The relative enthalpies at 0 K (ΔH_0 in eV) of reaction intermediates and products are given relative to reactants. The enthalpy at 0 K equals the sum of the potential energy and the zero-point vibrational energy. The black line and red line represent the triplet and quintet potential energy surfaces, respectively, and the superscripts indicate the spin multiplicities.

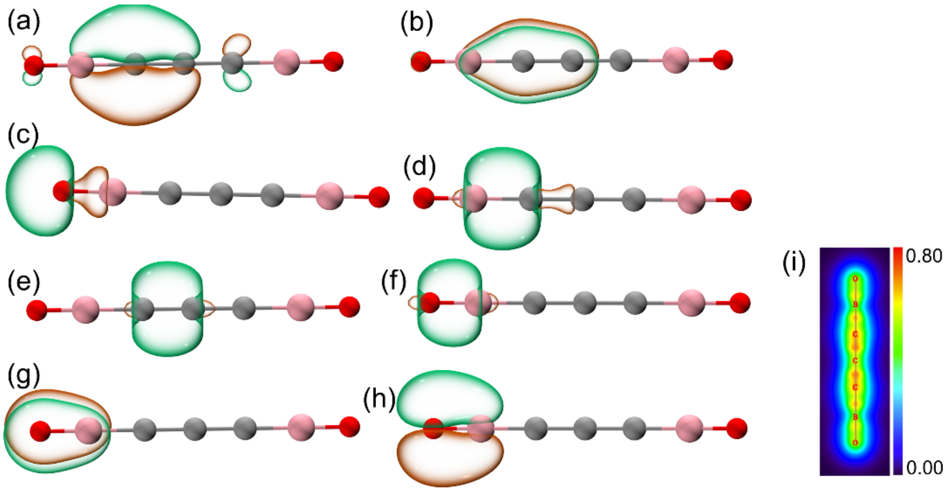

Figure S11. (a-h) Natural localized molecular orbitals and (i) π-electron localized orbital locator (LOL- π) for $C_3B_2O_2^-$. A large LOL-π value means that a region is dominated by a single localized π orbital that is large and extremal, and in the applications in the present study it is a covalent-bond indicator.

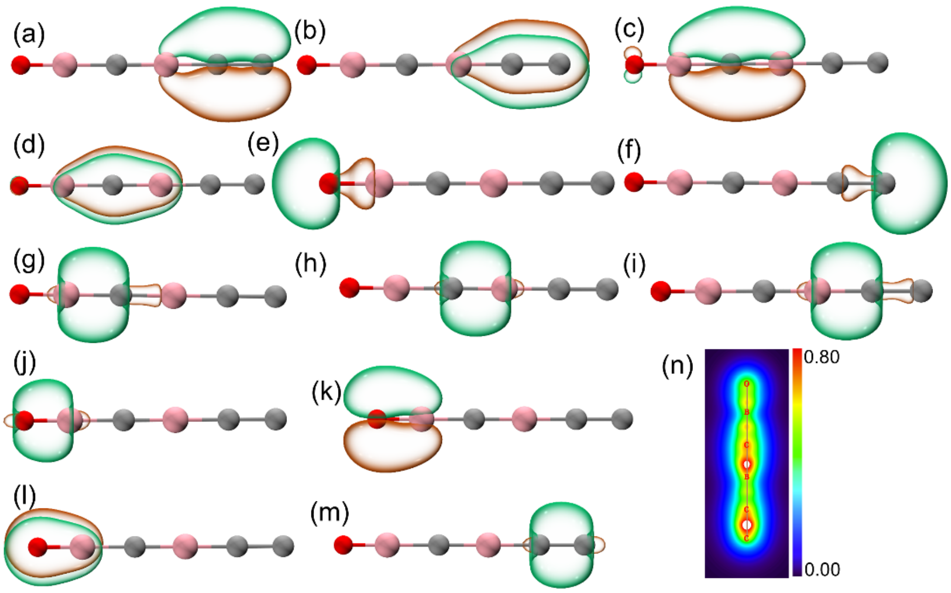

Figure S12. (a-m) Natural localized molecular orbitals and (n) LOL-π for $C_3B_2O^-$. See the caption of Figure S10 for an explanation of LOL-π.

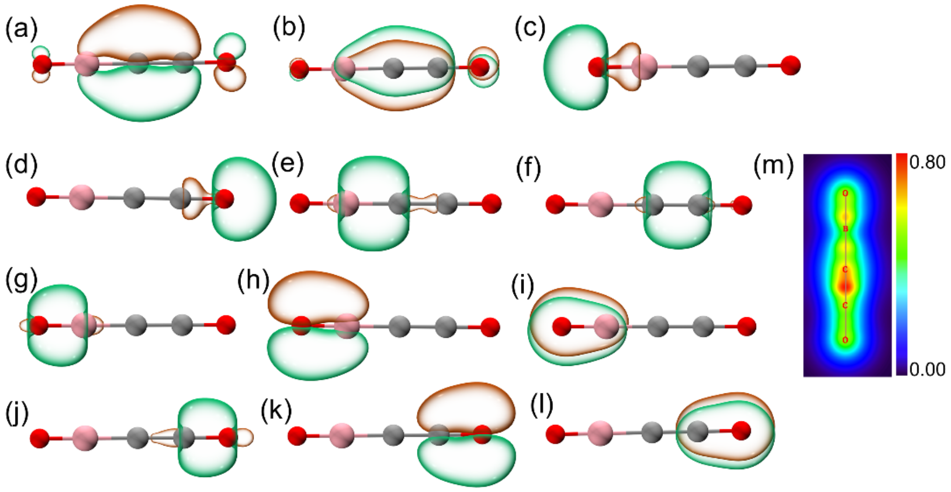

Figure S13. (a-l) Natural localized molecular orbitals and (m) LOL-π for $C_2BO_2^-$. See the caption of Figure S10 for an explanation of LOL-π.

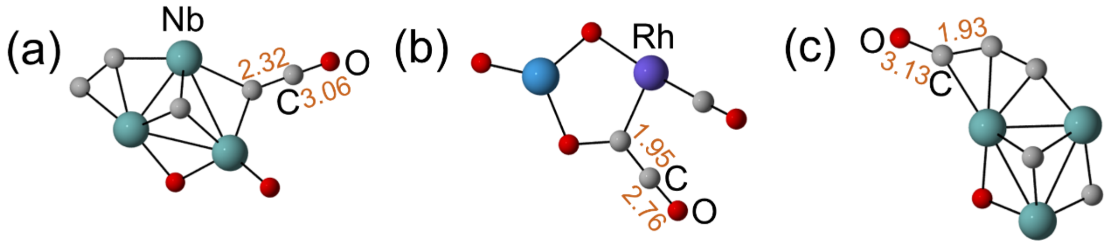

Figure S14. The key intermediates in (a) $Nb_3C_4O^-/CO_2$, (b) $RhTaC_2O_3^-/CO_2$, and (c) $Nb_3C_4^-/CO_2$ systems. The Wiberg bond indices are indicated.

4. References

[1] Y.-X. Zhao, Z.-Y. Li, Z. Yuan, X.-N. Li, S.-G. He, "Thermal Methane Conversion to Formaldehyde Promoted by Single Platinum Atoms in $PtAl_2O_4^-$ Cluster Anions." *Angewandte Chemie International Edition* **126**, (2014): 9636−9640.

[2] Z. Yuan, Z.-Y. Li, Z.-X. Zhou, Q.-Y. Liu, Y.-X. Zhao, S.-G. He, "Thermal Reactions of $(V_2O_5)_nO^-$ (n = 1−3) Cluster Anions with Ethylene and Propylene: Oxygen Atom Transfer Versus Molecular Association." *Journal Of Physical Chemistry C* **118**, (2014): 14967−14976.

[3] Z.-Y. Li, Z. Yuan, X.-N. Li, Y.-X. Zhao, S.-G. He, "CO Oxidation Catalyzed by Single Gold Atoms Supported on Aluminum Oxide Clusters." *Journal of the American Chemical Society* **136**, (2014): 14307−14313.

[4] Q.-Y. Liu, L.-R. Hu, Z.-Y. Li, C.-G. Ning, J.-B. Ma, "Photoelectron Imaging Spectroscopy of MoC^- and NbN^- Diatomic Anions: A Comparative Study." *Journal of Chemical Physics* **142**, (2015): 164301.

[5] M. J. Frisch, G. W. Trucks, H. B. Schlegel, G. E. Scuseria, M. A. Robb, J. R. Cheeseman, G. Scalmani, V. Barone, B. Mennucci, G. A. Petersson, et al, Gaussian, Inc., (Wallingford, CT), 2013.

[6] S. Grimme, J. Antony, S. Ehrlich, H. Krieg, "A Consistent and Accurate ab Initio Parametrization of Density Functional Dispersion Correction (DFT-D) for the 94 Elements H-Pu." *Journal of Chemical Physics* **132**, (2010): 154104.

[7] Z.-L. Wang, T.-T. Chen, W.-J. Chen, W.-L. Li, J. Zhao, X.-L. Jiang, J. Li, L.-S. Wang, H.-S. Hu, "The Smallest 4*f*-Metalla-Aromatic Molecule of *Cyclo*-PrB_2^- with Pr−B Multiple Bonds." *Chemical Science* **13**, (2022): 10082−10094.

[8] X. Chen, T.-T. Chen, W.-L. Li, J.-B. Lu, L.-J. Zhao, T. Jian, H.-S. Hu, L.-S. Wang, J. Li, "Lanthanides with Unusually Low Oxidation States in the PrB_3^- and PrB_4^- Boride Clusters." *Inorganic Chemistry* **58**, (2019): 411−418.

[9] D. A. Pantazis, F. Neese, "All-Electron Scalar Relativistic Basis Sets for the Lanthanides." *Journal of Chemical Theory and Computation* **5**, (2009): 2229−2238.

[10] B. Chen, G. L. Gutsev, D. Li, K. Ding, "Structure and Chemical Bonding in Medium-Size Boron Clusters Doped with Praseodymium." *Inorganic Chemistry* **61**, (2022): 7890−7896.

[11] R. Krishnan, J. S. Binkley, R. Seeger, J. A. Pople, "Self-Consistent Molecular Orbital Methods. XX. A Basis Set for Correlated Wave Functions." *Journal of Chemical Physics* **72**, (1980): 650−654.

[12] T. Clark, J. Chandrasekhar, G. W. Spitznagel, P. V. R. Schleyer, "Efficient Diffuse Function-Augmented Basis Sets for Anion Calculations. III. The 3-21+G Basis Set for First-Row Elements, Li–F." *Journal of Computational Chemistry* **4**, (1983): 294−301.

[13] M. Dolg, H. Stoll, H. Preuss, "Energy-Adjusted ab-initio Pseudopotentials for the Rare Earth Elements." *Journal of Chemical Physics* **90**, (1989): 1730−1734.

[14] X. Cao, M Dolg, "Segmented Contraction Scheme for Small-Core Lanthanide Pseudopotential Basis Sets." *Journal of Molecular Structure: Theochem* **581**, (2002): 139−147.

[15] J. Zhang, M. Dolg, "ABCluster: The Artificial Bee Colony Algorithm for Cluster Global Optimization." *Physical Chemistry Chemical Physics* **17**, (2015): 24173−24181.

[16] J. Zhang, M. Dolg, "Global Optimization of Clusters of Rigid Molecules Using the Artificial Bee Colony Algorithm." *Physical Chemistry Chemical Physics* **18**, (2016): 3003−3010.

[17] P. v. R. Schleyer, C. Maerker, A. Dransfeld, H. Jiao, N. J. R. v. E. Hommes, "Nucleus-Independent Chemical Shifts: A Simple and Efficient Aromaticity Probe." *Journal of the American Chemical Society* **118**, (1996): 6317−6318.

[18] R. Herges, D. Geuenich, "Delocalization of Electrons in Molecules." *The Journal of Physical Chemistry A* **105**, (2001): 3214−3220.

[19] H. L. Schmider, A. D. Becke, "Chemical Content of the Kinetic Energy Density." *Journal of Molecular Structure: Theochem* **527**, (2000): 51−61.

[20] H. L. Schmider, A. D. Becke, "Two Functions of the Density Matrix and Their Relation to the Chemical Bond." *Journal of Chemical Physics* **116**, (2002): 3184−3193.

[21] T. Lu, F. Chen, "Multiwfn: A Multifunctional Wavefunction Analyzer." *Journal of Computational Chemistry* **33**, (2012): 580−592.

[22] D. Y. Zubarev, A. I. Boldyrev, "Developing Paradigms of Chemical Bonding: Adaptive Natural Density Partitioning." *Physical Chemistry Chemical Physics* **10**, (2008): 5207−5217.

[23] E. D. Glendening, F. Weinhold, “Natural Resonance Theory: II. Natural Bond Order and Valency.” *Journal of Computational Chemistry* **19**, (1998): 610−627.

[24] E. D. Glendening, C. R. Landis, F. Weinhold, “NbO 6.0: Natural Bond Orbital Analysis Program.” *Journal of Computational Chemistry* **34**, (2013): 1429−1437.

[25] D. W. Szczepanik, M. Andrzejak, J. Dominikowska, B. Pawelek, T. M. Krygowski, H. Szatylowicz, M. Sola, “The Electron Density of Delocalized Bonds (EDDB) Applied for Quantifying Aromaticity.” *Physical Chemistry Chemical Physics* **19**, (2017): 28970−28981.

[26] D. W. Szczepanik, M. Andrzejak, K. Dyduch, E. Zak, M. Makowski, G. Mazur, J. Mrozek, “A Uniform Approach to the Description of Multicenter Bonding.” *Physical Chemistry Chemical Physics* **16**, (2014): 20514−20523.

[27] C. Angeli, S. Borini, M. Cestari, R. Cimiraglia, “A Quasidegenerate Formulation of the Second Order-Electron Valence State Perturbation Theory Approach.” *Journal of Chemical Physics* **121**, (2004): 4043−4049.

[28] L. Lang, K. Sivalingam, F. Neese, “The Combination of Multipartitioning of the Hamiltonian with Canonical Van Vleck Perturbation Theory Leads to A Hermitian Variant of Quasidegenerate n-Electron Valence Perturbation Theory.” *Journal of Chemical Physics* **152**, (2020): 014109.

[29] L. Lang, M. Atanasov, F. Neese, “Improvement of ab Initio Ligand Field Theory by Means of Multistate Perturbation Theory.” *The Journal of Physical Chemistry A* **124**, (2020): 1025−1037.

[30] F. Neese, “The ORCA Program System.” *Wiley Interdisciplinary Reviews-computational Molecular Science* **2**, (2012): 73−78.

[31] F. Neese, F. Wennmohs, U. Becker, C. Riplinger, “The ORCA Quantum Chemistry Program Package.” *Journal of Chemical Physics* **152**, (2020): 224108.

[32] F. Weigend, R. Ahlrichs, “Balanced Basis Sets of Split Valence, Triple Zeta Valence and Quadruple Zeta Valence Quality for H to Rn: Design and Assessment of Accuracy.” *Physical Chemistry Chemical Physics* **7**, (2005): 3297−3305.

[33] Y.-Q. Ding, Z.-Y. Chen, F.-X. Zhang, J.-B. Ma. “Coupling of N_2 and O_2 in the Gas Phase to Synthesize Nitric Oxide at Room Temperature: A Zeldovich-Like Strategy.” *Journal of Physical Chemistry Letters* **14**, (2023): 7597−7602.
